# Editor's comments

**DOI:** 10.4103/0971-9261.54815

**Published:** 2009

**Authors:** Minu Bajpai

**Affiliations:** Guest Editor

In this study, Spot VCUG under intermittent fluoroscopy was done by the traditional technique. No attempt was made to record the volume of contrast injected at the time of recording the status of reflux during VCUG. The same was done for DRC. Therefore, the results are not comparable. The data of DRC results from the present study simply endorse the known position on VCUG.

The authors admit that grading of reflux is not possible with radionuclide studies. This is of paramount importance. In McLaren and Simpson's report (BJU 2001; 87:93-97), 50% of the low grades of reflux were missed by DRC. Besides other information, the authors had concluded that DRC and VCUG both should be carried out to improve upon picking up reflux and grading it. Although, the authors of the present study also conclude the same, the methodology is biased towards DRC. The data can be compared only when the same bladder filling criteria are applied to both modalities of investigations. And, if applied to VCUG alone, more information can be generated vis a vis DRC. The latter would then become redundant.

